# Extensive Field Survey, Laboratory and Greenhouse Studies Reveal Complex Nature of *Pseudomonas syringae*-Associated Hazelnut Decline in Central Italy

**DOI:** 10.1371/journal.pone.0147584

**Published:** 2016-02-03

**Authors:** Jay Ram Lamichhane, Claudia Bartoli, Leonardo Varvaro

**Affiliations:** 1 Department of Science and Technology for Agriculture, Forestry, Nature and Energy (DAFNE), Tuscia University, Viterbo, Italy; 2 Hazelnut Research Center, Viterbo, Italy; 3 INRA, UAR 1240 Eco-Innov, BP 01, Thiverval-Grignon, France; 4 INRA, Laboratoire des Interactions Plantes-Microorganismes (LIPM), UMR441, Castanet-Tolosan, France; 5 CNRS, Laboratoire des Interactions Plantes-Microorganismes (LIPM), UMR2594, Castanet-Tolosan, France; University of the West of England, UNITED KINGDOM

## Abstract

*Pseudomonas avellanae* (Pav) has been reported as the causal agent of bacterial decline and bacterial canker of hazelnut in Italy and Greece, respectively. Both hazelnut diseases were reported to be similar in terms of symptoms, severity and persistence. In this study, we found that both symptomatic and asymptomatic trees in the field were colonized by Pav. Multilocus Sequence Typing (MLST) analysis showed that Pav strains isolated during this study in Italy belong to the *P*. *syringae* phylogroup 1 and they are closely related to Pav strains previously isolated in Greece from hazelnut bacterial canker. On the other hand, strains isolated in earlier studies from hazelnut decline in Italy belong to both phylogroup 1 and 2 of *P*. *syringae*. Both phylogroup 1 strains of *P*. *syringae* from Greece and Italy are different than strains isolated in this study in terms of their capacity to excrete fluorescent pigments on different media. Despite the same plant genotype and cropping practices adopted, the incidence of hazelnut decline ranged from nearly 0 to 91% across our study sites. No disease developed on plants inoculated with Pav through wounding while leaf scar inoculations produced only mild disease symptoms. Based on our results and the previously reported correlation between pedo-climatic conditions and hazelnut decline, we conclude that hazelnut decline in central Italy could be incited by a combination of predisposing (adverse pedo-climatic conditions) and contributing factors (Pav). Because this is a true decline different from “bacterial canker” described in Greece, we refer to it as hazelnut decline (HD).

## Introduction

Diseases caused by *Pseudomonas syringae* on woody plants are of increasing concern because of their potential to rapidly spread worldwide with severe economic losses [1]. For example, bacterial canker of kiwifruit has rapidly spread worldwide within a short period of time [2]. Likewise, bleeding canker of horse chestnut has quickly established itself as a major threat to horse chestnut throughout Northwest Europe [3]. However, numerous diseases caused by *P*. *syringae* are also reported only once from a specific area without any further reports elsewhere. Examples are bacterial leaf spot of coffee [4], citrus blast of orange and mandarin [5], bacterial canker of amurmaackia [6], bacterial dieback and canker of poplar [7], bacterial leaf spot of Japanese fig [8] and bacterial leaf disease of kohekohe [9]. This explains that the occurrence and spread of *P*. *syringae* diseases may be affected by several factors.

Since the late 1980’s, orchards of hazelnut (*Corylus avellana*) in central Italy faced a sudden dieback phenomenon called “Moria” (hereafter referred to as hazelnut decline, HD) [10–12]. The symptoms consisted of rapid wilting of twigs, branches or the entire trees [12]. At the time, the phenomenon was reported only from a small area of just a few hectares, and the disease appeared mainly during late spring and summer. The decline, however, spread rapidly in the following years, affecting thousands of hectares throughout the Province of Viterbo, central Italy [12,13]. For many years, the researchers involved in understanding the disease aetiology did not isolate a common causal agent but different ones. This led them to suppose that the disease could be of a complex origin [10]. Consequently, different factors/parasites/pathogens were reported as the causal agent of the decline such as nematodes [14] and bacteria belonging to the “true Erwinia” group [12].

A few years before the appearance of hazelnut decline in Italy, a similar bacterial disease on hazelnut was reported also from northern Greece. The symptoms described from Greek orchards were, to some extent, similar to those observed in central Italy [15]. Symptoms such as the presence of deep cankers along branches and trunks and the formation of white bacterial exudates on infected trunks, during the advanced phase of the infection, however, were described only from Greece [15]. After nearly ten years, the causal agent of the disease in Greece was identified as *Pseudomonas avellanae* (Pav) and the disease was named bacterial canker [16]. Subsequently, Scortichini and Tropiano [17] reported the presence of Pav also in central Italy suggesting that Pav was responsible for hazelnut decline throughout Italian orchards. Only nearly after three decades, phylogenetic studies based on strains from Greece showed that they belong to both *P*. *syringae* phylogroup 1 and 2 [18] which subsequently led to the hypothesis of the convergent evolution of the two genotypes on hazelnut [19].

While hazelnut bacterial canker did not persist in the cultivated areas in Greece, hazelnut decline (HD) is still a problem for hazelnut growers in central Italy. Over three thousand hectares of hazelnut orchards have been abandoned in the province of Viterbo since its first appearance in 1987. HD was very severe in some orchards, but milder in others [20–22]. Surprisingly, no further spread of HD symptoms was reported beyond the areas concerned by the initial foci. Of nearly 20 000 ha of intensive hazelnut farming of central Italy [23], about 20% is affected by HD. It is intriguing that the decline remained confined within limited areas without affecting neighboring orchards. Recent studies [24,25] reported that plant genotypes and cultural practices in central Italy are almost homogeneous which suggests that these factors do not have an important role in HD. Earlier studies, however, found a positive correlation between pedo-climatic factors such as annual rainfall, frost events, soil nitrogen content and HD, suggesting that unfavorable pedo-climatic conditions for plant growth, at least in some of the sites studied, were responsible for HD [21,22].

The objectives of this study were to investigate: i) whether or not HD incidence was different across our study sites, ii) if Pav was present in orchards without HD symptoms, iii) if Pav alone was able to cause HD, as severe as those observed in the field, in artificially inoculated conditions, and iv) whether strains from this study were phylogenetically related to the strains previously isolated from hazelnut.

## Materials and Methods

### Ethics Statement

No specific permits were required for the described field studies. At each study site, the landowner granted us permission to collect hazelnut samples. The studies carried out during the consecutive years 2010–2012 did not involve endangered or protected species. Field surveys were made across the hazelnut orchards, in the Province of Viterbo, central Italy while laboratory and green house studies were carried out at Tuscia University.

### Study site and data collection

Each municipality had a different number of study sites (from 1 to 6) based on the orchards’ extension that varied from 5 to 25 ha. The experimental design used for the survey was similar as described previously [24] except for the fact that we performed the study on a smaller scale and used a lower number of plants per year. From each site (total 29), 300 hazelnut trees (100 per year) were randomly surveyed for HD symptoms from early spring to early autumn. The same trees were considered for all three years. The description of the study sites has been published [24]. Information on the surveyed areas, average plant age and cultivars are presented in S1 Table. During field surveys, attention was paid to verify the presence of previously described hazelnut decline symptoms from central Italy [17] and bacterial canker symptoms reported from northern Greece [15].

### Sample collection and bacterial isolation

**Fig 1:**
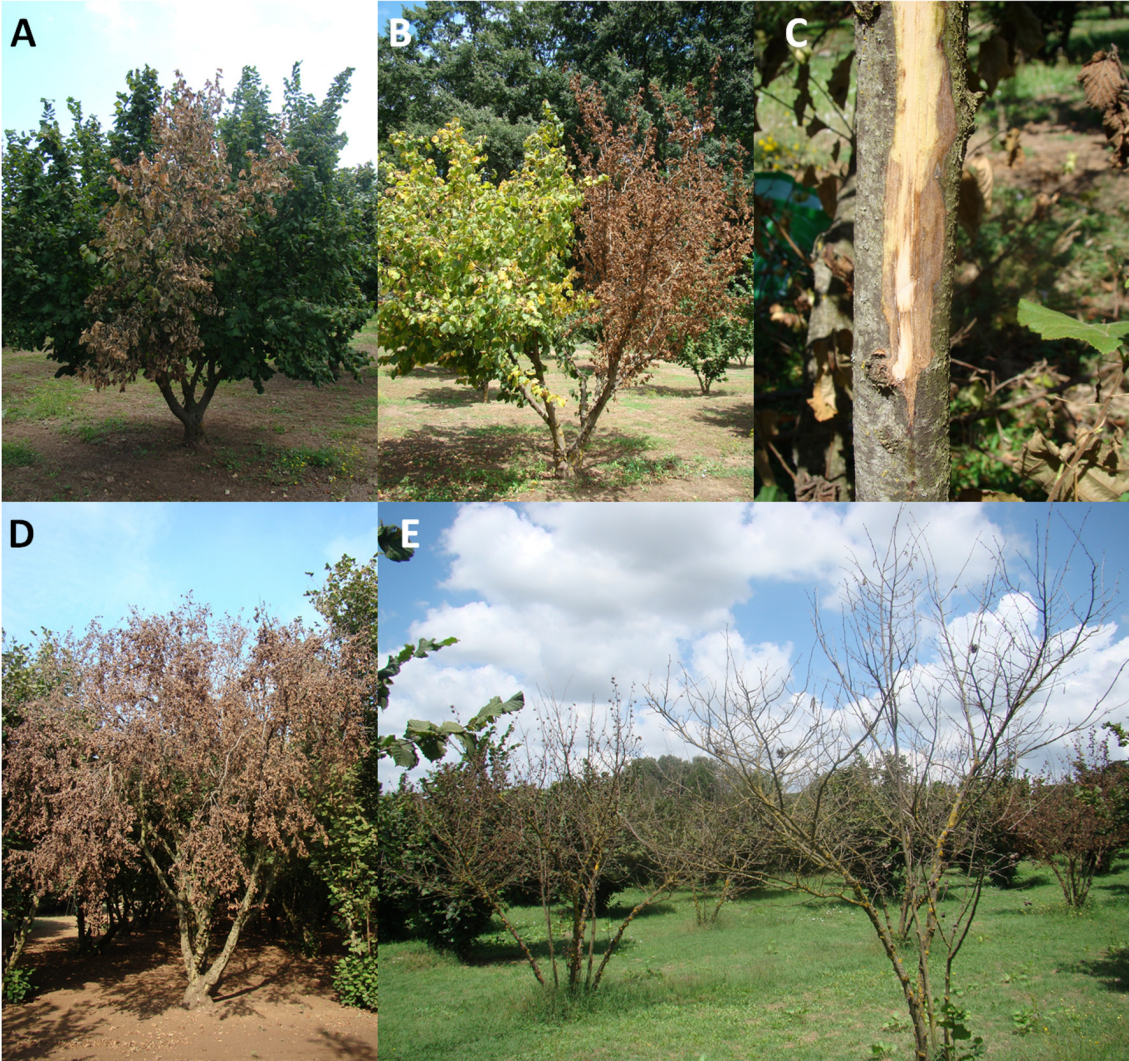
Characteristic hazelnut decline symptoms observed on hazelnut plants (cv. Tonda Gentile Romana). Decline began with sudden wilting of a stem (A) while the rest was still intact. Successively, decline affected the whole branch which manifested the dieback while the remaining branch manifested suffering state with pale green foliage (B). When the bark of branch bearing pale green foliage was excised, discoloration of cambium could be observed (C). During the end of vegetative phase complete plant death occurred although leaves were still attached (D). The following year, also the plants present in the proximity of the plant that showed initial decline symptoms appeared completely affected (E).

**Fig 2:**
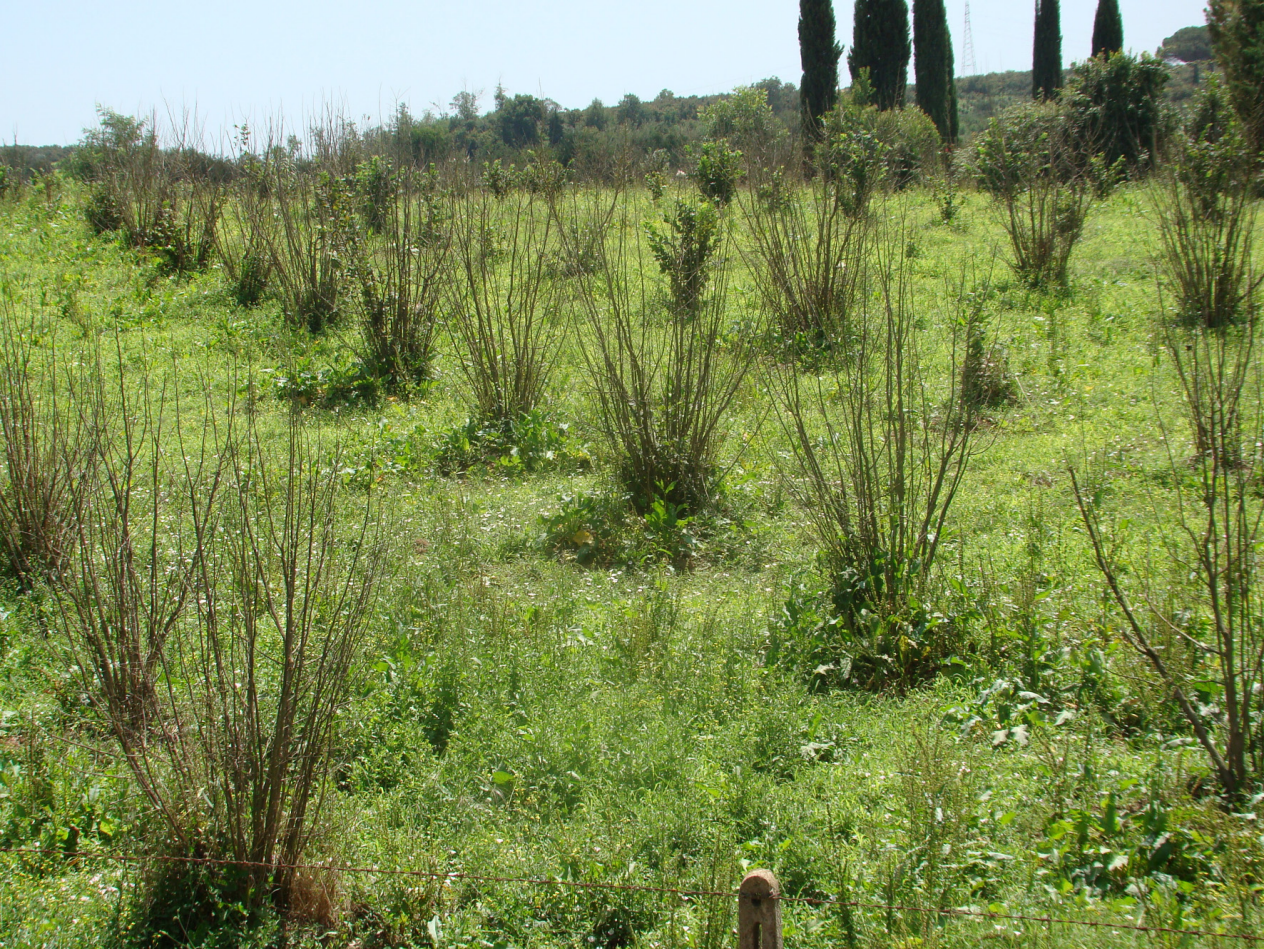
A very young hazelnut plantation (4 year old plants, cv. Tonda gentile Romana) completely decimated by hazelnut decline phenomenon. The young plants were planted in the same plot where severely affected old plants (15-year-old) were removed.

Hazelnut twigs were collected between March and August for each year. Plant parts showing HD symptoms (Figs 1 and 2), as those reported in the literature [15,17], were cut, put separately into sterile plastic lab bags and brought to the laboratory. To avoid the contamination of samples with the cutting tools, the latter were sterilized, between one sampling and another, in copper solution for 2 min followed by two 1 min rinses in sterile distilled water [24]. In addition, asymptomatic hazelnut twigs from trees affected by HD were also collected from the study sites. Overall, the number of samples taken from each site ranged from 5 to 30, depending on the presence and the incidence of HD. In fields where the symptoms were absent, only a few samples were collected within an approximate radius of 100 m. By contrast, samples were collected from several neighboring plants when the incidence was high. This sampling scheme was adopted to obtain a sufficient number of bacterial isolates from symptomatic plant tissues within a given site in order to investigate possible phenotypic and genetic diversity among the bacterial strains [24].

Isolations were made as follows: the twigs were cut into *ca*. 10 cm pieces, washed in running tap water and surface-sterilized as described previously [26]. The pieces were washed in 70% ethanol for 5 min, followed by washings in 1% NaOCl for 1 min followed by three rinses in sterile distilled water. The bark was removed with a sterile scalpel and the pieces were cut into lengths of *ca*. 1 cm which were crushed in sterile mortars containing sterilized distilled water. In case of symptomatic twigs, the tissue used for isolation was excised between apparently healthy and diseases parts. A loopful of the resulting suspension was streaked on nutrient agar supplemented with 5% sucrose (NAS) and cycloheximide (100 ppm). The latter was used to prevent fungal contamination. Plates were incubated at 26±1°C and examined until bacterial growth was observed. Bacterial colonies resembling Pav were re-streaked to obtain single colonies on the same medium. Only cytochrome oxidase-negative strains were further characterized as *P*. *syringae* is cytochrome oxidase-negative [27].

### Hazelnut decline incidence

Plants from each site were geographically referenced by the Universal Transverse Mercator coordinate system with a handheld Global Positioning system instrument. The instrument used was a Garmin III Plus (Garmin International Inc., Olathe, KS). Geographical referencing was performed in order to identify the same plants in the following year. Any symptoms suggestive to bacterial canker described from Greece [15] and hazelnut decline reported from central Italy [17] were taken into consideration. The incidence of HD was calculated by the proportion of diseased plants within the total [28].

### Characterizations of bacterial isolates

#### Phenotypic characterization

The detection of Pav-like bacterial colonies was performed by using the levan-positive morphology on NAS medium as described previously [29]. Overall, three bacterial isolates morphologically resembling to the reference strains of Pav—including levan positive feature—from all 29 but one sites (total 87 isolates), were selected for phenotypic characterization. Two reference Greek strains of Pav (the type strain: NCPPB 3487 = PavBPIC631 and NCPPB 3489) were also used as positive controls. The isolates were tested for the production of fluorescent pigment on KB [30], CSGA [31] and PGS agar [32], LOPAT (levan, oxidase, potato rot, arginine dihydrolase and tobacco hypersensitive reaction), GATTa (Gelatine liquefaction, Aesculin hydrolysis, Tyrosinase activity and Tartrate utilization), use of different carbon sources (glycerol, D-mannitol, D-sorbitol, glucose, fructose, maltose), type of metabolism (fermentative or oxidative), growth on L-Arginin and L-Asparagin, productions of indole, lecithinase, urease and H2S from peptone, arbutin and starch hydrolysis, minimum and maximum growth temperatures, tolerance to salt (2 and 5% salt) and ice nucleation activity (INA). All tests were performed according to the methods described previously [33] except INA test which was performed according to Lindow [34].

#### Multilocus sequence typing analysis

In order to investigate whether isolates randomly chosen from the study sites were identical or different, BOX PCR was performed on their pure colonies as described previously [35]. Subsequently, a number of representative isolates from the study sites were further analyzed by sequencing the *cts*, *gapA*, *gyrB* and *rpoD* housekeeping genes. Primer pairs used and PCR conditions were the same as previously described [36]. PCR products obtained were sequenced with Macrogen Europe and sequences were deposited in GenBank http://www.ncbi.nlm.nih.gov/genbank (Accession numbers KJ621601 to KJ621644). The housekeeping gene sequences were aligned and trimmed to the same size with DAMBE version 5 [37]. Sequences representative of different *P*. *syringae* phylogroups—including sequences of the *P*. *syringae* strains isolated both in Greece and Italy [18]—were added to the MLST analysis. All the MLST sequences and the relative phylogroup and pathovar affiliations of the strains are reported in S3 Table. Bayesian tree were constructed with the Mr. Bayes program [38] using 500000 generations burned in 50.

#### Hypersensitivity reaction tests

Hypersensitivity reaction tests were performed on lemon fruits and bean pods purchased from the supermarket as described previously [39]. The inoculum consisted of aqueous suspensions of 48h bacterial cultures from KB plates adjusted to 1x10^8^ CFU ml^–1^. For each isolate, two lemon fruits and bean pods were inoculated. A 10μl drop of bacterial suspension was placed on each lemon fruit and bean pod previously pricked with a sterile hypodermic needle. All inoculated materials were placed in a plastic box containing moist papers to maintain the humidity and incubated for 3 days at ambient temperature. The appearance of brownish necrotic halo/spot was considered as positive reaction.

#### Pathogenicity tests

Pathogenicity tests were performed on two-year-old, healthy pot-cultivated hazelnut plants (cv. Tonda Gentile Romana; the most predominant in the field). Bacterial suspensions of the same concentration as described above were prepared. Ten bacterial isolates obtained during this study were used (S2 Table). The inoculations were made in the late spring by using wound and leaf scar inoculation techniques. Wound inoculation [24] was made by placing a 10μl drop of bacterial suspension under small flaps created under the bark with a sterile scalpel. The wounds were then bound with parafilm strips which were removed after 48 hours. Leaf scar inoculation was made by removing leaf petiole with a razor blade on the attached point of the stem where the same quantity of bacterial suspension was applied. Here, the parafilm was not used since the suspension was rapidly absorbed by a negative pressure formed in the plant vascular system. For each isolate and for each kind of inoculation, 4 replicates were made and the experiment was repeated twice. The reference strains NCPPB 3487, NCPPB 3489 and sterile distilled water were used as positive and negative controls, respectively, on the same number of plants. The plants were maintained in a greenhouse at ambient temperature (between 18 to 23°C) until symptom development. Re-isolations were carried out 8 weeks post-inoculation. Prior to processing the samples, the severity of the disease was assessed by measuring both the length of external and internal necrosis developed on the inoculated plants.

### Statistical analysis

Data from the artificial inoculation tests were not normally distributed and for that reason they were analyzed using the non-parametric Wilcoxon signed-rank test employing R code. Values of P≤0.05 were considered to be statistically significant.

## Results

### Hazelnut decline symptoms and incidence in the field

Initial symptoms appeared in early spring which consisted in failure to bud break, bud withering or the retardation in sprouting. In early summer, sudden wilting of one or more stems within a branch appeared (Fig 1A). Whole branch dieback, located at the initial infection site, could be observed in late summer (Fig 1B). The remaining branch of the infected plant had pale green foliage (Fig 1B). A light brown discoloration of the cambium (Fig 1C) could be noted following the excision of the bark of twigs bearing pale green foliage. Apparently, the progression of decline symptoms halted by late autumn. However, previously affected plants were often found completely dead in the following year by the end of the vegetative season (Fig 1D). No bacterial exudates or canker symptoms were observed on plants affected by HD. The blighted leaves remained attached on the plant even after the fall of healthy senescent leaves in autumn (Fig 1D).

**Fig 3:**
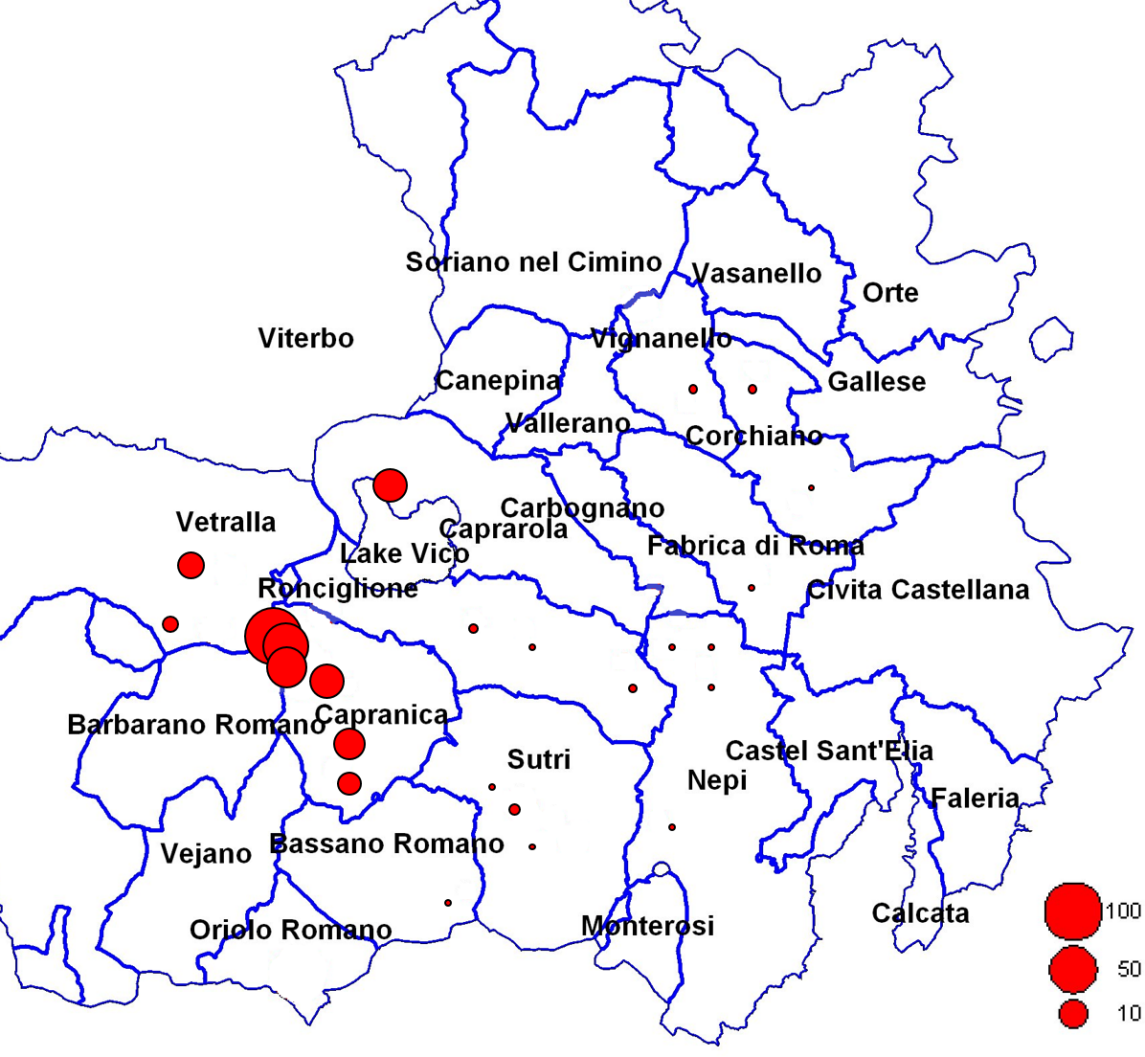
Incidence of hazelnut decline (%) across the study sites in the province of Viterbo. The circle size inside the map indicates the different hazelnut decline incidence.

Even though HD symptoms rarely manifested in the majority of sites observed, the decline was a severe problem within a few specific sites including in newly planted orchards—where the old plants severely affected by HD were previously removed (Fig 2). Of 29 sites investigated, the average HD incidence was nearly zero in 20 sites while it was low in 4 sites with values that ranged from 7 to 15%. By contrast, a high to very high incidence of HD was found in the remaining 5 sites with values that ranged from 22 to 91% (S1 Table; Fig 3).

### Isolation of Pav from hazelnut twigs

Overall, Pav was recovered both from symptomatic and asymptomatic twigs collected over the three years of study (Table 1). While Pav was successfully isolated from symptomatic twigs collected from ten out of eleven municipalities (except in Fabrica di Roma), its isolation from asymptomatic twigs was successful only from seven out of eleven municipalities. In particular, symptomatic twigs collected from study sites with a high HD incidence (Capranica, Caprarola, Vetralla and Ronciglione) yielded higher percentage of Pav (Table 1; S4 Table). However, rather than a low HD incidence, both symptomatic and asymptomatic twigs sampled in Soriano yielded a high percentage of Pav (Table 1; S4 Table).

**Table 1:** Isolation of *Pseudomonas avellanae* (Pav) from hazelnut twigs collected from 2010 to 2012. Samples were collected from March to August, each year. The average percentage of Pav isolation for the three years is calculated by the ratio between the number of Pav-positive samples and the total number of samples used for isolation.

Municipality	Avergae disease incidence (%)	Avergae % of Pav isolation from^1^	
		Symtomatic tissues	Asymptomatic tissues
Capranica	39.27	81.42	3.89
Caprarola	12.00	35.75	10.00
Vetralla	10.50	74.98	3.33
Ronciglione	10.25	48.60	5.00
Soriano	0.66	61.90	26.67
Sutri	0.66	22.49	4.44
Vignanello	0.66	15.63	6.67
Bassano Romano	0.33	20.59	0.00
Corchiano	0.33	6.35	0.00
Fabrica di Roma	0.33	0.00	0.00
Nepi	0.33	19.68	0.00

^1^Asymptomatic twigs were sampled from plants affected by hazelnut decline.

### Characterizations of bacterial isolates

#### Phenotypic characterization and MLST analysis

After 2 days post incubation, the colonies of potential Pav isolates formed circular, domed, pearl-white levan positive colonies of 2–2.5 mm in diameter on NAS medium, resembling those of reference Pav strains. All phenotypic characteristics of Pav resembling colonies obtained in this study were similar to those previously isolated strains from hazelnut affected by bacterial canker in Greece, except for their capacity to produce fluorescence pigment on KB, CSGA and PGS agar media (S5 Table).

**Fig 4:**
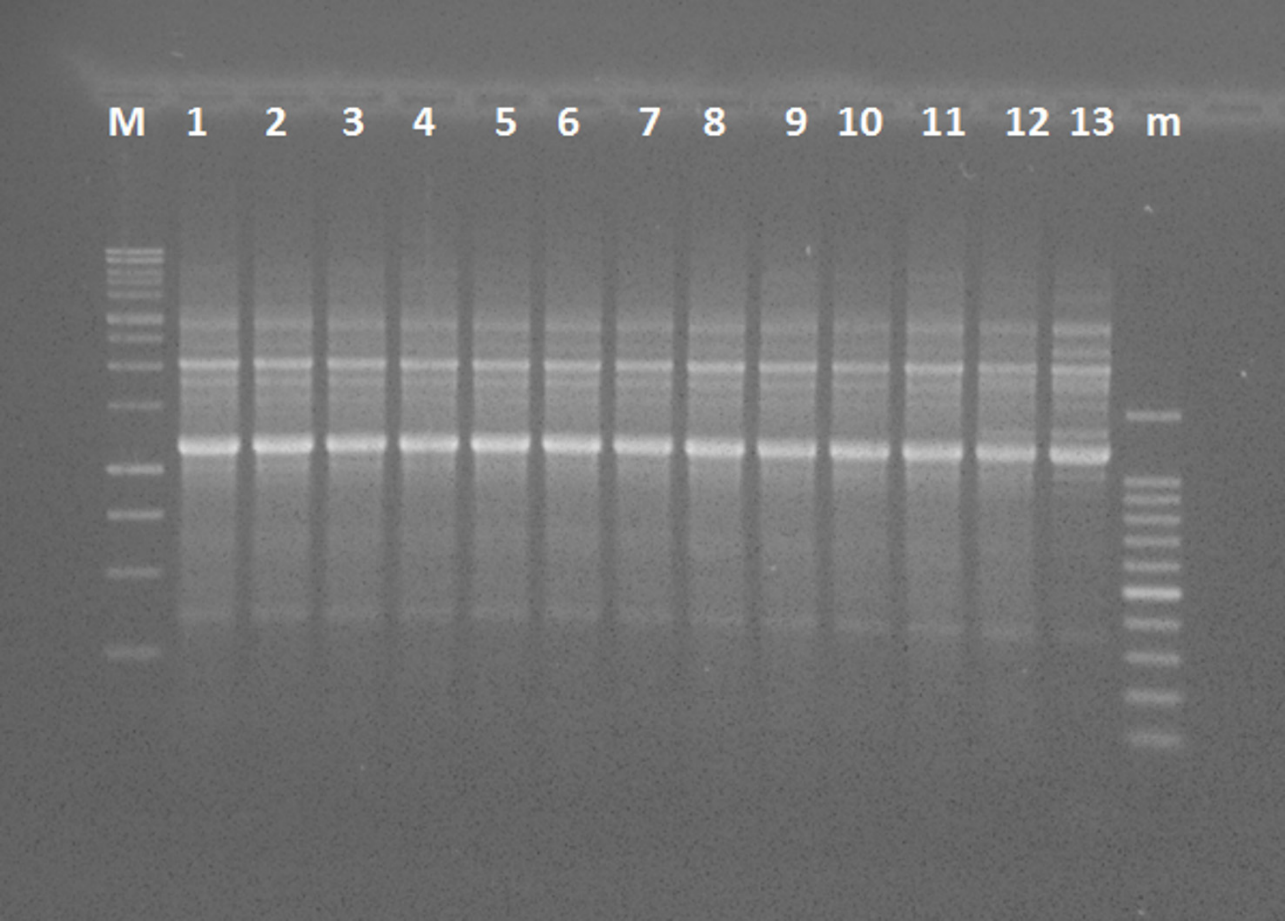
An example of BOX PCR fingerprint patterns obtained for genomic DNAs of *Pseudomonas avellanae* strains isolated from *Corylus avellana* by using the BOX primer set. Lanes from left to right—M: molecular size marker (1-kb ladder), 1: strain PaVT1, 2: strain PaVT2, 3: strain PaVT5, 4: strain PaVT6, 5: strain PaVT8, 6: strain PaVT10, 7: strain PaVT17, 8: strain PaVT24, 9: strain PaVT25, 10: strain PaVT28, 11: strain PaVT29, 12: strain NCPPB 3489, 13: strain NCPPB 3487 and m: molecular size marker (100bp ladder). All strains isolated in this study were clonal and no diversity was observed among them.

**Fig 5:**
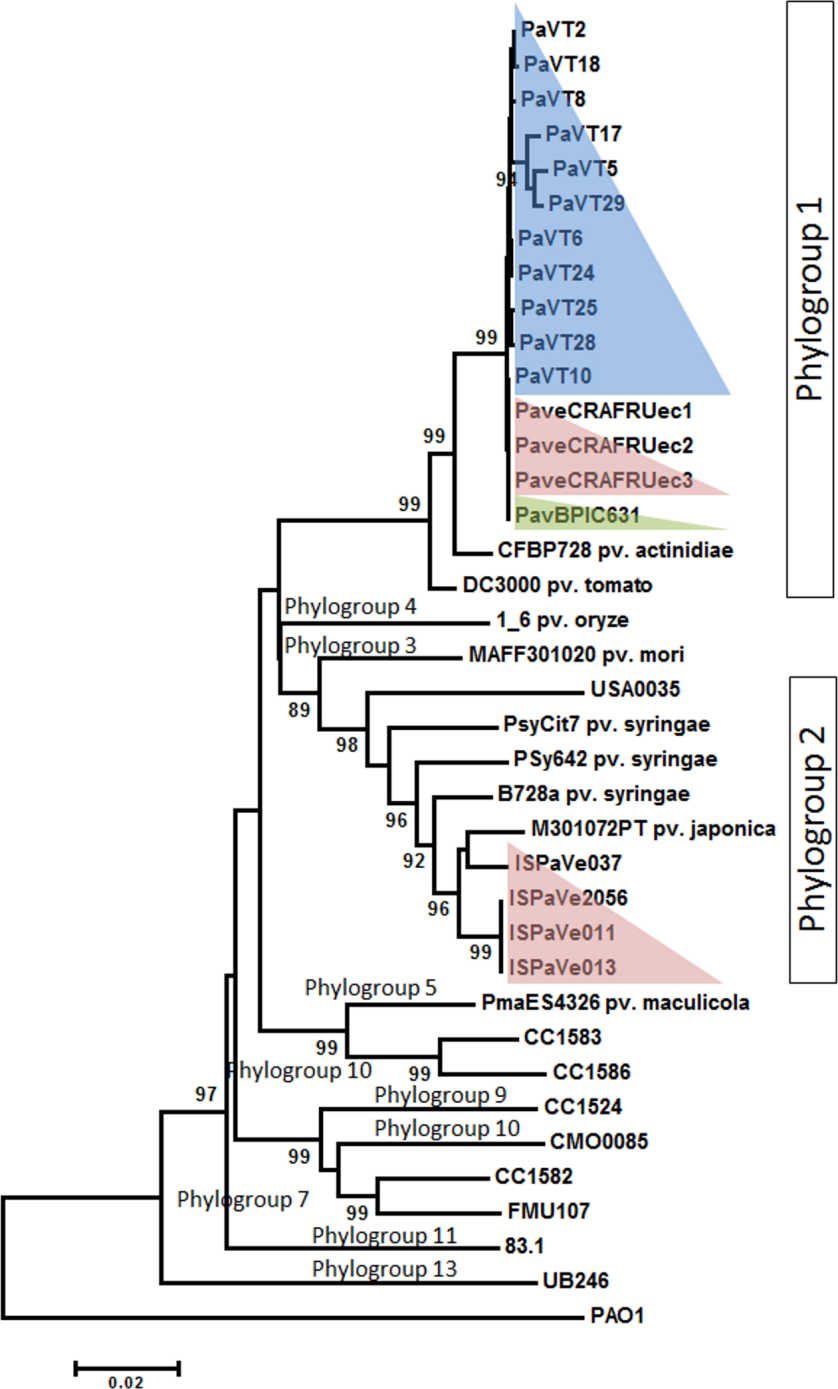
Bayesian tree constructed on the concatenated sequences of *cts*, *gyrB*, *rpoD* and *gapA* (1465bp). Bootstrap values are indicated at each node, while names at the branches indicate the name of the strains. Pathovar affiliations are reported for some of the *Pseudomonas syringae* strains belonging to different phylogenetic groups. Sequences for the different *P*. *syringae* strains and their phylogroup affiliation were described previously by Berge et al. [46]. MLST sequences of the Pav strains isolated previously from Greece and Italy were reported by Wang et al. [18]. Sequences for PavCRAFRUec1, PavCRAFRUec2 and PavCRAFRUec3 are published by Marcelletti and Scortichini [41]. *Pseudomonas aeruginona* (PAO1) was used as an outgroup. Triangles with different colours are used to label strains in the following way. Blue triangles: strains isolated in this study, red triangles: strains isolated in central Italy during the first epidemic, and green triangles: strains isolated in Greece.

BOX PCR performed on randomly selected levan positive isolates showed no diversity among the isolates obtained in this study. An example of BOX PCR fingerprint patterns obtained for genomic DNAs of isolates obtained in this study is shown in Fig 4 together with the reference strains of *P*. *avellanae* isolated in Greece. Because the fingerprinting of the isolates were identical, only eleven of them were chosen for MLST analysis which confirmed that all of them belong to the *P*. *syringae* phylogroup 1 (Figs 5 and S1). All the strains were closely related, in terms of phylogeny, to the strains isolated in Greece (NCPPB 3487 = PavBPIC631 and PavBPIC641) and to some of the strains isolated from previous epidemics in Italy (PavCRAFRUec1, PavCRAFRUec2, PavCRAFRUec3, PavISH1, PavISH2, PavISH3, PavISH4). Interestingly, strain PaVT10 was closely related, based on the MLST analysis, to strains previously isolated from Greek and Italian hazelnut disease epidemics. On the other hand, no strains isolated from this study were placed into the *P*. *syringae* phylogroup 2 (Figs 5 and S1) confirming that only strains of the *P*. *syringae* phylogroup 1 were associated with HD observed in this study.

#### Hypersensitivity reaction and pathogenicity tests

**Fig 6:**
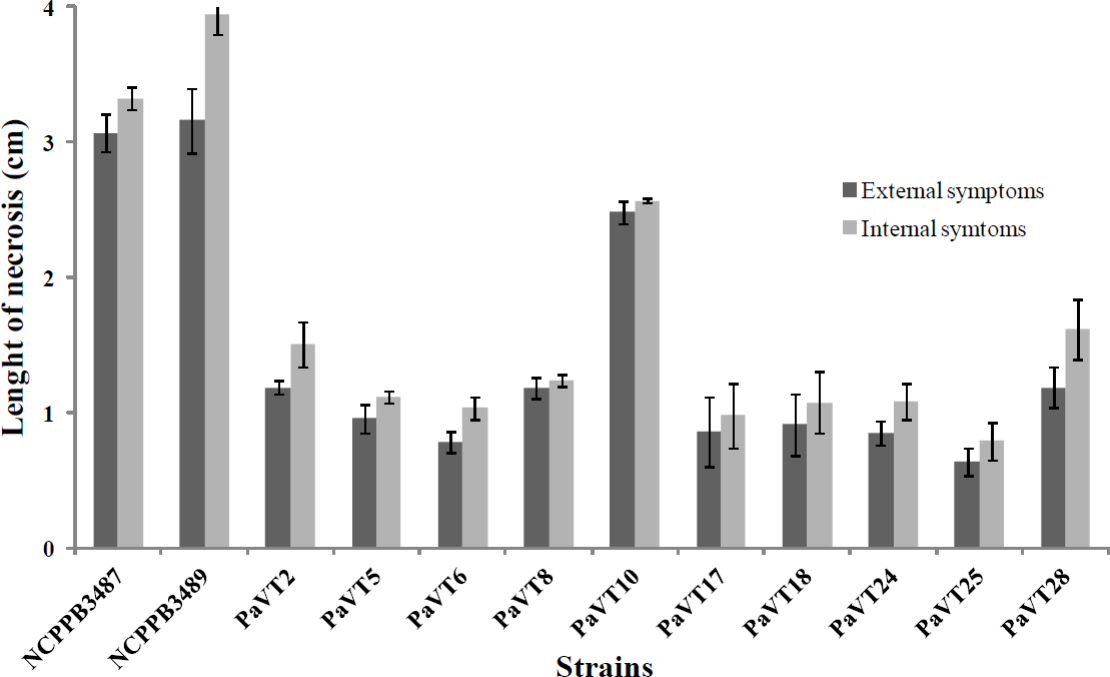
Length of external and internal necroses developed on hazelnut plants (cv. Tonda Gentile Romana) inoculated through leaf scar. The length was measured after 4 weeks post infection. The reference strains of *Pseudomonas avellanae* (NCPPB 3487 (= BPIC631) and NCPPB 3489) previously isolated in Greece from bacterial canker symptoms produced mild external and internal necroses, together with the strain PaVT10 isolated in this study. The rest of the strains tested in this study caused only weak necrosis on the inoculated plants. The error bars represent the standard error of the measurements.

None of the isolates or the reference strains caused lesions on lemon fruit and bean pods. In contrast, small necrosis around the inoculated site was observed after 4 weeks post infection on hazelnut plants inoculated through leaf scar (S2 Table). While the reference strains of Pav produced mild external and internal necroses, together with the isolate PaVT10 (the only phylogroup 1 strains isolated in this study which is clonal to previously isolated strains), the rest of the isolates caused only weak necrosis on the plants (Fig 6). The plants inoculated with sterile distilled water remained healthy.

While considering the aggressiveness of the strains in causing external necrosis, no differences statistically significant (P = 0.7181) were found between the reference strains of Pav. By contrast, differences statistically significant (P ≤ 0.05) were observed between the reference strains and most of the isolates obtained in this study. There were also differences statistically significant (P ≤ 0.05) among the isolates obtained in this study with a few exceptions (S2 Table).

The severity of internal necrosis caused on hazelnut plants differed significantly among the strains/isolates tested with a few exceptions (Fig 6; S2 Table). While no differences significantly different (P = 0.9895) were found between the reference strains, there were differences statistically significant (P ≤ 0.05) between the reference strains of Pav and all isolates obtained in this study. Similarly, we found statistically different significant (P ≤ 0.05) among some isolates recovered in this study. Overall, the two reference strains of Pav and the isolate PaVT10 caused mild internal necrosis while the other strains caused only weak necrosis on internal plant tissues (Fig 6; S2 Table).

No symptom developed on wound inoculated plants. In this case, oxidation of the woody tissues around the wound, due to the bark incision, produced light browning which could be exchanged for false positive symptoms. The excision of the underlying cambium tissues however could provide the evidence of the real disease symptoms, given by the internal necrosis. Indeed, none of the plants showed internal necrosis when the bark was excised. Nonetheless, both symptomatic (leaf scar inoculation) and asymptomatic (wound inoculation) plants yielded bacteria from the inoculated point and had the same characteristics of the inoculated strains.

## Discussion

The strains of *P*. *syringae* associated with hazelnut bacterial canker in Greece and HD in central Italy have been the object of continuous changes in terms of taxonomic classification. An earlier study [16], based on the phenotypes of strains isolated from Greece, proposed strains isolated from bacterial canker as being part of *P*. *syringae* species complex (the so-called pv. *avellanae*). A following study [40] based on DNA-DNA hybridization—which included both the strains isolated from Greece and central Italy—indicated that these strains were very much apart from all pathovars of *P*. *syringae* including the so-called pv. *syringae* and proposed them as a different species, *P*. *avellanae*. Subsequent studies based on the MLST analysis and whole genome sequencing revealed that strains previously isolated in Greece belonged to the *P*. *syringae* phylogroup 1 while strains isolated in Italy belonged to the phylogroup 2 of *P*. *syringae* [18,41]. Further studies based on comparative genomics also hypothesized that the two phylogenetic *P*. *syringae* lines experienced a convergent evolution that led to a similar pathogenic phenotype on hazelnut plants [19,41]. However, here we demonstrate that only the phylogroup 1 strains of *P*. *syringae* are associated with currently observed hazelnut plants affected by HD in central Italy.

Differences in disease in terms of occurrence, incidence, severity and spread in our study sites clearly suggest that HD is a complex disease. This explains the fact that a classical disease triangle—interactions among the host, the pathogen and the favorable environmental conditions—is sufficient for some *P*. *syringae* diseases to develop but not for others. The contrasting examples of disease spread between HD and bacterial canker of kiwifruit [42] is a telling example in this regard. Indeed, the presence of only *P*. *syringae* phylogroup 1 strains found in this study on the one hand and the previous description about the two divergent phylogroup on the other are the confirmation that HD is a complex disease. The presence of severe *P*. *syringae* disease on hazelnut has been reported only from Greece and Italy although the range of hazelnut cultivations widely exceeds that of kiwifruit [43]. Here, we did not investigate on the relationship between the incidence of HD and pedo-climatic factors. However, previous studies [21,22] based on the geostatistics confirmed that HD occurs in areas where pedo-climatic conditions are unfavorable for plant growth. In particular, spring frost events that predispose plant to stress were positively correlated with HD. In addition, rainfall and soil nitrogen were reported to foster bacterial survival in plant tissues and consequent attacks. Other studies confirmed similar relationships while studying other pathosystems in the same study sites [24,25].

While Pav strains isolated in this study are non-fluorescent Pav from Greece are fluorescent. This suggests that although they are phylogenetically close to each other at the 4 MLST loci, there are in fact phenotypic differences that could explain why the Greek strains incite canker but the Italian strains do not. Alternative explanations could be particular pedo-climatic conditions, cultivars used in Italy or absence of other bacteria in Italy that do not allow for the development of cankers. Possibly, other bacterial microbiota present only in Greece are necessary for canker development. Previously, only fluorescent Pav populations were described from hazelnut with HD symptoms in Italy [17]. There could be three possible explanations for this finding. First, no field study and bacterial isolations were carried out in the last decade although the decline is still severe throughout some study areas. Hence, it is possible that Pav populations have undergone a phenotypic change, losing the capacity of fluorescent pigment production. Indeed, strains previously isolated from central Italy were reported to lose fluorescence after a number of transfers on media which was influenced by subculture on NAS medium [44]. Second, Pav populations, reported as the causal agent of bacterial canker, was first identified in Greece and all of them were fluorescent [15]. Therefore, authors from central Italy, at the time, might have considered only the fluorescent bacterial populations underestimating the non-fluorescent ones. Third, the bacteria isolated in this study are not descended from those isolated ten years ago. In particular, a combination of the second and third hypotheses seems to be the most plausible explanation as it seems that there has been a replacement of phylogroup 2 strains of *P*. *syringae* with those of phylogroup 1.

One of the most important findings of this study is that the inoculation technique plays a critical role in symptom development. Indeed, of wound and leaf scar inoculation techniques used at the same conditions and with the same inoculum concentration, only the leaf scar inoculation resulted effective. This could be mainly due to the importance of ports of entry as opportunistic pathogens, such as *P*. *syringae*, could not always incite disease symptoms if sensitive ports of entry are not used. Psallidas [16] from Greece reported, however, that wound inoculation allowed to obtain canker symptoms although the author did not mention about the severity of symptoms developed. Artificial inoculations with Pav on hazelnut performed in this study caused only mild symptoms which did not reflect HD symptoms under field conditions. The lack of severe disease development, even with a high concentration of the inoculum used, further indicates the complexity of HD. Previously, Scortichini and Lazzari [45] reported a very limited systemic movement of Pav in the plant vascular system under experimental inoculation in the field. This also raises questions on the exclusive role of Pav in the occurrence of HD. Therefore, HD seems to be of a complex etiology although it is difficult to demonstrate the interactions between the host, predisposing and contributing factors under experimental conditions.

Despite permanent HD problems, growers in central Italy continuously tend to replant new hazelnut plants within the same orchards by replacing affected plants with the healthy one. However, attempts to replant young hazelnut plants have been often unsuccessful. Although the newly planted young plants seem to have normal growth for a certain period of time, they are subsequently subject to decline symptoms. Hence, a better soil management combined with adequate cultural practices—that improve crop health and avoid stress conditions—could be a viable solution for those specific sites where HD remains a perennial problem. Another solution could be to grow the crop less intensively in those areas where plants often suffer due to unfavorable pedo-climatic conditions.

## Supporting Information

S1 Fig Bayesian tree constructed on the concatenated sequences of gyrB, rpoD and gapA (1049bp; see the Fig 4 legend for detailed information). (XLSX)

S1 Table Study areas, hazelnut cultivars, plant age and hazelnut decline incidence across the Viterbo province. (DOCX)

S2 Table Results of pathogenicity tests on hazelnut. (XLSX)

S3 Table Multilocus Sequence Typing analysis sequences used to compare Pav strains. (XLSX)

S4 Table Number of Pav isolates obtained from the study sites. (XLSX)

S5 Table Results of the phenotypic tests. (XLSX)
