# Supplementary material for: Extensive Field Survey, Laboratory and Greenhouse Studies Reveal Complex Nature of Pseudomonas syringae-Associated Hazelnut Decline in Central Italy
Source: PLoS One. 2016 Feb 3;11(2):e0147584. doi: 10.1371/journal.pone.0147584 (PMC4739619; doi:10.1371/journal.pone.0147584)
Supplement: S1 Table — (DOCX) [file pone.0147584.s002.docx]

**Table S1.** Study areas, hazelnut cultivars, plant age and sudden hazelnut decline incidence across the Viterbo province

| **Municipality** | **Site** | **Cultivar^a^** | **Plant average age** | **Average incidence (%)^b^** |
| --- | --- | --- | --- | --- |
| Sutri | 1 | TGR, N | 35 | <1 |
|  | 2 | TGR, N | 30 | <1 |
|  | 3 | TGR, TDG | 25 | <1 |
| Ronciglione | 1 | TGR, N | 25 | 15 |
|  | 2 | TGR, N, TDG | 30 | <1 |
|  | 3 | TGR, N | 30 | <1 |
|  | 4 | TGR, N | 30 | 39 |
| Vetralla | 1 | TGR, TDG | 15 | 7 |
|  | 2 | TGR, N | 25 | 14 |
| Capranica | 1 | TGR, N | 30 | 10 |
|  | 2 | TGR, N | 20 | 24 |
|  | 3 | TGR, N | 4 | 46 |
|  | 4 | TGR, N | 15 | 42 |
|  | 5 | TGR, N | 15 | 91 |
|  | 6 | TGR, N | 25 | 22 |
| Nepi | 1 | TGR, N | 25 | <1 |
|  | 2 | TGR, N | 30 | <1 |
|  | 3 | TGR, N | 20 | <1 |
|  | 4 | TGR, N, TDG | 20 | <1 |
|  | 5 | TGR, N | 25 | <1 |
| Vignanello | 1 | TGR, N, TDG | 25 | <1 |
| Soriano | 1 | TGR, N | 12 | <1 |
| Caprarola | 1 | TGR, N | 25 | 23 |
|  | 2 |  | 25 | <1 |
| Bassano Romano | 1 | TGR, N | 25 | <1 |
| Corchiano | 1 | TGR | 25 | <1 |
|  | 2 | TGR, TDG | 25 | <1 |
|  | 3 | TGR, TDG | 25 | <1 |
| Fabrica di Roma | 1 | TGR, N | 30 | <1 |
| Tonda Gentile Romana (TGR), Nocchione (N), Tonda Di Giffoni (TDG) | | | | |
| ^a^Only TDG represents over 85% of the entire cultivation | | | | |
| ^b^Values are the mean of three years and calculated by the proportion of diseased plants within the total | | | | |
